# Attention-Deficit/Hyperactivity Disorder, Racial Disparities, and Involvement in the Juvenile Justice System: A Narrative Review

**DOI:** 10.7759/cureus.108669

**Published:** 2026-05-11

**Authors:** Justin B Atkins, Oscar A Toro Ruilowa, Deanna Chea, Caitlin Chen

**Affiliations:** 1 Medical School, University of Nevada, Las Vegas School of Medicine, Las Vegas, USA; 2 Psychiatry and Behavioral Health, University of Nevada, Las Vegas School of Medicine, Las Vegas, USA

**Keywords:** adhd, delinquency, health disparities, incarceration, juvenile justice, pediatric mental health, treatment access

## Abstract

Attention-deficit/hyperactivity disorder (ADHD) is a common neurodevelopmental condition associated with adverse behavioral, social, and legal outcomes across childhood and adolescence. Untreated ADHD is associated with increased involvement in the criminal justice system, while racial and ethnic disparities in diagnosis and treatment may contribute to inequitable outcomes among minority youth. This narrative review examines the relationships among ADHD, pharmacologic treatment, juvenile justice involvement, and racial/ethnic disparities affecting minority youth. A targeted review of peer-reviewed literature published between 2000 and 2026 was conducted using PubMed and Google Scholar, including cohort studies, longitudinal analyses, meta-analyses, and nationally representative datasets, to assess ADHD prevalence, associations with delinquency and criminal justice outcomes, treatment effects, and disparities in diagnosis and care. Across studies, ADHD is consistently associated with increased rates of police contact, court involvement, criminal convictions, incarceration, and shorter time to re-offense. Pharmacologic treatment is associated with reduced rates of criminal convictions and substance-related problems. Nevertheless, racial and ethnic disparities persist, with minority youth significantly less likely to receive a diagnosis or treatment despite a similar symptom burden. ADHD is also substantially overrepresented in justice-involved populations, with approximately 25-30% of individuals meeting diagnostic criteria. ADHD represents a potentially modifiable factor along pathways to juvenile justice involvement, although outcomes are shaped by both clinical and structural determinants. Expanding equitable screening, improving access to longitudinal pharmacologic and behavioral care, strengthening coordination among healthcare, educational, and community systems, and integrating mental health services within juvenile justice settings may reduce downstream justice system involvement among vulnerable youth populations.

## Introduction and background

Attention-deficit/hyperactivity disorder (ADHD) is one of the most common neurodevelopmental disorders across childhood and adolescence, with global prevalence estimates ranging from approximately 5% to 10%, depending on diagnostic methods and population characteristics [1,2]. ADHD is characterized by developmentally impairing patterns of inattention, hyperactivity, and impulsivity that may persist across the lifespan and affect academic performance, social functioning, emotional regulation, occupational stability, and health outcomes into adulthood [3]. Current pediatric guidelines emphasize comprehensive diagnostic evaluation, assessment for comorbid conditions, and longitudinal treatment planning, underscoring ADHD as a persistent public health concern [4,5]. Increasingly, ADHD is recognized not only as a clinical condition but also as a factor influencing trajectories related to education, public safety, and criminal justice involvement.

Core features of ADHD, including impulsivity, impaired behavioral inhibition, and executive dysfunction, may contribute to risk-taking, poor decision-making, and difficulty conforming behavior to social or institutional expectations [6]. Longitudinal research demonstrates that ADHD symptoms often persist into adulthood, reinforcing the importance of early identification and sustained treatment planning [7]. More broadly, deficits in self-control during childhood have been associated with adverse outcomes across health, economic, and public safety domains, suggesting that behavioral dysregulation may carry long-term consequences beyond the clinical setting [8].

A growing body of evidence links ADHD with delinquency and criminal justice involvement. Meta-analytic and longitudinal data demonstrate associations between ADHD and increased risk of criminal behavior, substance-related problems, and social impairment [9]. Population-based analyses have shown higher rates of police contact, court involvement, criminal convictions, and incarceration among individuals with ADHD compared with peers without ADHD [10]. Longitudinal follow-up studies suggest that ADHD may influence the trajectory and recurrence of offending behavior over time [11]. In large registry-based studies, ADHD has been associated with criminal convictions, while periods of pharmacologic treatment have been associated with lower rates of criminality [12].

ADHD is also associated with increased mortality across children, adolescents, and adults, reflecting the broader clinical and public health significance of the disorder beyond behavioral outcomes alone [13]. ADHD is substantially overrepresented in correctional and detention settings. Meta-analytic evidence suggests that approximately one-quarter of incarcerated individuals meet criteria for ADHD, a prevalence several times higher than that observed in the general population [14,15]. Studies of incarcerated adults suggest that ADHD symptoms may be associated with personality traits and behavioral patterns relevant to institutional functioning and recidivism risk [16]. Among detained youth, psychiatric disorders are highly prevalent, frequently comorbid, and often underrecognized before justice system involvement [17,18].

The pathway from ADHD symptoms to justice involvement is not purely individual. Structural and institutional factors influence how behavioral symptoms are interpreted and managed. School disciplinary systems may serve as an early point of institutional contact when disruptive or impulsive behaviors are addressed through exclusionary or punitive responses rather than clinical assessment and support [19]. These experiences intersect with broader patterns of disproportionate minority contact, in which minority youth experience higher rates of surveillance, referral, detention, and deeper system involvement [20].

Racial and ethnic disparities in ADHD diagnosis and treatment further complicate this relationship. Minority youth, including Black and Hispanic children, are less likely to receive ADHD diagnoses than White peers despite comparable symptom burden [21]. National data demonstrate persistent disparities in treatment access and continuity of care [22,23]. Sociodemographic factors, including race, ethnicity, socioeconomic status, and healthcare access, consistently shape ADHD identification and management [24]. In this context, underdiagnosed or untreated ADHD may increase vulnerability to academic failure, school discipline, and subsequent justice system involvement.

Treatment is a central component in understanding the relationship between ADHD and justice outcomes. Evidence-based pharmacologic treatment improves core symptoms and functional outcomes [25]. Population-based studies suggest that ADHD treatment may be associated with reductions in criminality and substance-related problems, a key pathway linking ADHD with adverse legal outcomes [26,27]. These findings suggest that early identification and sustained treatment may influence behavioral trajectories, educational stability, and justice system exposure.

This narrative review synthesizes current evidence on the relationship between ADHD, treatment, and juvenile justice involvement, with particular emphasis on racial and ethnic disparities in diagnosis and care. The review focuses on ADHD prevalence, developmental mechanisms, criminal justice outcomes, treatment effects, psychiatric comorbidity among justice-involved youth, school and institutional pathways, and disparities that may influence patterns of clinical intervention and system involvement.

Methodology

A targeted narrative review of peer-reviewed literature was conducted to evaluate the relationships among ADHD, pharmacologic treatment, racial and ethnic disparities, and criminal justice outcomes in youth and young adult populations. A narrative approach was selected due to substantial heterogeneity in study designs, exposure definitions, and outcome measures across the relevant literature, which precluded quantitative meta-analysis. The objective was to integrate evidence across psychiatry, pediatrics, psychology, criminology, public health, and juvenile justice research.

A structured literature search was performed using PubMed and Google Scholar in March 2026. The following representative Boolean search string was used in PubMed: (“ADHD” OR “attention-deficit/hyperactivity disorder”) AND (“juvenile justice” OR “delinquen” OR “criminal”** OR “incarcerat*” OR “detention” OR “recidivism”) AND (“racial disparities” OR “ethnic disparities” OR “school discipline” OR “disproportionate minority contact”).

Google Scholar was searched using combinations of the above terms. Due to known variability in Google Scholar search results, searches were limited to the first 200 results per query to improve consistency. Additional studies were identified through manual review of reference lists from key articles. The search included studies published from January 1, 2000, through March 2026, with the inclusion of selected foundational studies published before 2000 where relevant, such as Barkley, 1997. Only English-language, peer-reviewed articles were included.

Studies were eligible for inclusion if they met one or more of the following criteria: (1) addressed ADHD prevalence, persistence, or clinical features; (2) examined associations between ADHD and delinquency or criminal justice outcomes; (3) evaluated ADHD treatment effects on behavioral, substance-related, or justice-related outcomes; (4) assessed racial or ethnic disparities in ADHD diagnosis or treatment; or (5) examined institutional or structural factors relevant to juvenile justice involvement. Included study designs consisted of cohort studies, longitudinal analyses, case-control studies, cross-sectional studies, meta-analyses, systematic reviews, and clinical practice guidelines involving human subjects.

Exclusion criteria included: (1) case reports or case series with fewer than 10 participants; (2) editorials, commentaries, or letters without original data; (3) animal or in vitro studies; (4) conference abstracts without full-text publication; and (5) non-peer-reviewed sources, including dissertations and preprints.

Study selection and data extraction were conducted by all authors. Titles and abstracts were screened for relevance, followed by full-text review of potentially eligible studies. Discrepancies were resolved through discussion and consensus.

Given the heterogeneity across studies, findings were synthesized qualitatively rather than quantitatively. To strengthen methodological rigor, included studies were assessed for overall quality based on study design, sample size, and consistency with existing literature. Greater interpretive weight was given to large population-based studies, longitudinal analyses, and meta-analyses. Consistent findings across multiple higher-quality studies were emphasized, while isolated findings were interpreted with caution.

This structured narrative approach was selected to provide a clinically relevant and systems-informed synthesis of ADHD as both a neurodevelopmental condition and a factor associated with downstream justice system involvement.

## Review

ADHD and criminal justice involvement

Across multiple cohort and longitudinal studies, ADHD is consistently associated with an increased risk of criminal justice involvement. Population-based analyses demonstrate that individuals with ADHD have higher rates of police contact, court charges, criminal convictions, and incarceration compared with individuals without ADHD [9,10]. In a large national birth cohort study (N > 20,000), ADHD was associated with a significantly increased risk of interaction with the criminal justice system across multiple domains, including arrests and legal charges [10]. Similarly, longitudinal follow-up studies of incarcerated populations demonstrate that ADHD is associated with persistent patterns of offending behavior over time [11].

These findings are supported by broader meta-analytic data demonstrating an increased risk of criminal behavior and social impairment among individuals with ADHD [9]. Importantly, these associations appear to persist across developmental stages, reflecting both childhood-onset behavioral dysregulation and the persistence of ADHD symptoms into adulthood in a substantial proportion of individuals [7].

Emerging evidence suggests that this relationship is not uniform across all individuals with ADHD. Some studies indicate that hyperactive-impulsive symptom presentations may be more strongly associated with externalizing behaviors and justice system involvement than predominantly inattentive presentations. Additionally, greater symptom severity has been associated with a higher risk of adverse behavioral outcomes, suggesting a potential dose-response relationship. ADHD is associated with an increased risk of justice system involvement in both males and females, although differences in behavioral expression and referral patterns may influence how this risk manifests across populations.

**Figure 1:**
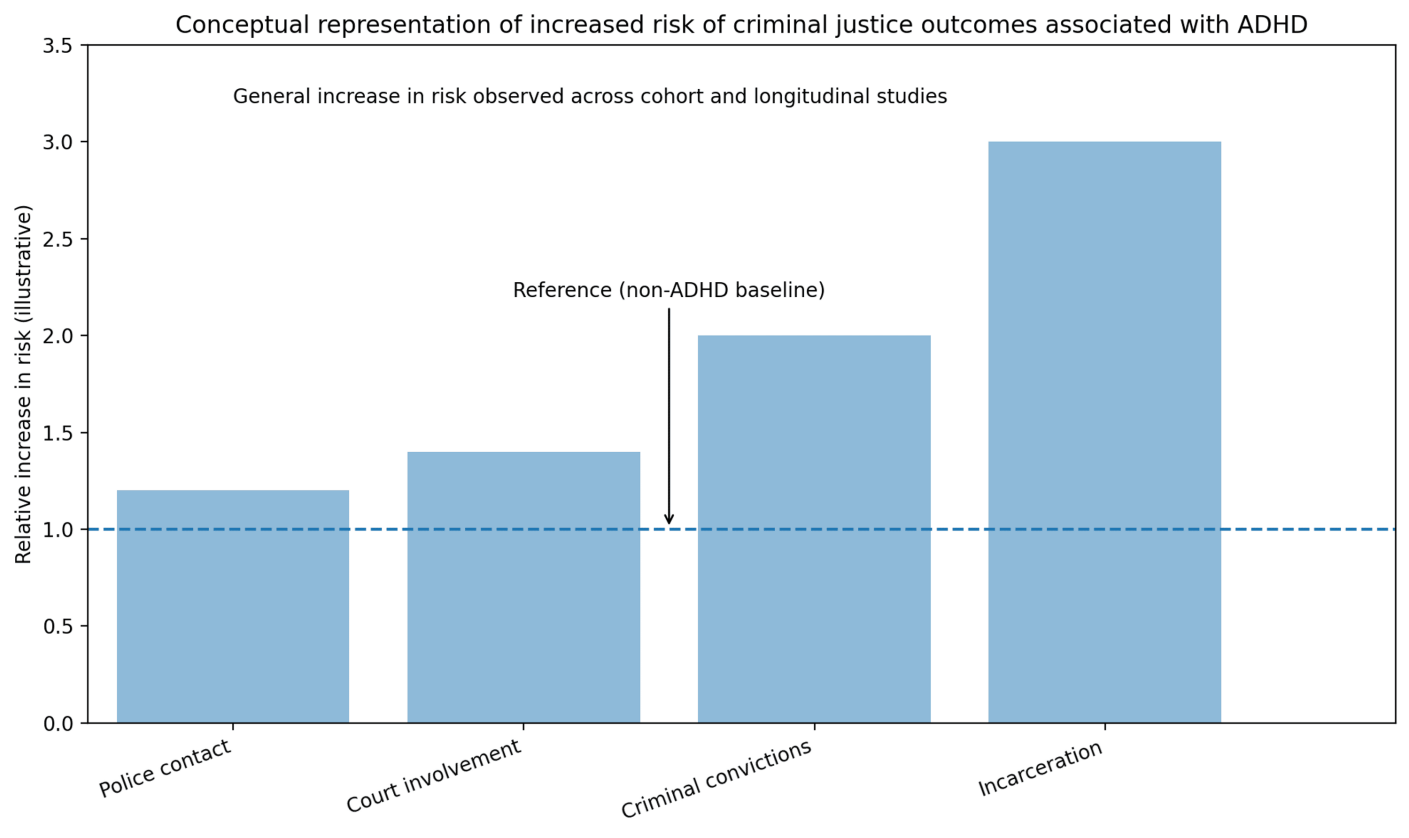
Conceptual representation of the increased risk of criminal justice outcomes associated with ADHD. This figure provides a conceptual illustration of the relative increase in the risk of police contact, court involvement, criminal convictions, and incarceration among individuals with ADHD compared with counterparts without ADHD. The visual representation is based on patterns consistently reported across cohort and longitudinal studies [9-11]. The values depicted are illustrative and do not represent pooled effect sizes, CIs, or estimates from a single dataset. The figure is intended to summarize general trends in the literature rather than provide precise quantitative comparisons. Image credit: Atkins JB. Created using Microsoft Excel and Google Slides. No generative AI tools were used in the creation of this figure. ADHD: Attention-Deficit/Hyperactivity Disorder.

Collectively, these data suggest that ADHD is associated with a sustained elevation in risk for justice system involvement rather than a transient or context-specific effect. These associations are illustrated in Figure 1.

Recidivism and behavioral trajectory

In addition to increased rates of initial justice system involvement, ADHD has been associated with differences in the timing and patterns of reoffending. Longitudinal analyses demonstrate that individuals with ADHD may experience a shorter time to reoffending compared with individuals without ADHD, suggesting a more rapid cycle of recidivism [11]. In long-term follow-up studies of incarcerated men with a 15-year follow-up period and a sample size of approximately 200, ADHD was associated with earlier and more frequent reoffending, indicating that ADHD may influence not only the likelihood of offending but also the pattern and persistence of criminal behavior over time [11].

These findings are consistent with theoretical models of ADHD that emphasize impaired behavioral inhibition, impulsivity, and difficulty with delayed reward processing as core mechanisms influencing decision-making and risk-taking behaviors [6]. From a developmental perspective, these impairments may contribute to repeated system contact in the absence of effective intervention, reinforcing a cycle of recidivism over time.

Impact of pharmacologic treatment

Pharmacologic treatment of ADHD has been associated with reductions in behaviors linked to criminal justice involvement. In large population-based registry studies (N > 25,000), periods during which individuals received ADHD medication were associated with lower rates of criminal convictions compared with non-treatment periods [12]. These findings have been observed across both male and female populations, suggesting that treatment effects are not limited to a single demographic group.

Additional longitudinal analyses further support these findings. ADHD medication has been associated with reductions in substance-related problems in large population-based cohorts (N > 150,000), which represent a key pathway linking ADHD with adverse legal and health outcomes [26,27]. Given the well-established association between substance use and criminal behavior, these findings suggest that treatment may influence justice system involvement through multiple behavioral pathways.

The mechanisms underlying these associations are not fully established but likely include improvements in impulse control and decision-making, reductions in substance use, and improvements in behavioral regulation and functional outcomes. Meta-analytic evidence also supports the efficacy and tolerability of pharmacologic treatment in improving core ADHD symptoms across populations [25]. While these studies were not designed specifically to evaluate criminal justice outcomes, the observed improvements in attention, impulse control, and behavioral regulation provide plausible pathways linking treatment to downstream outcomes.

However, all available evidence remains observational, and causal relationships cannot be definitively established. Unmeasured confounding factors, including socioeconomic context, family environment, and co-occurring conditions, may influence both treatment exposure and observed outcomes.

**Figure 2:**
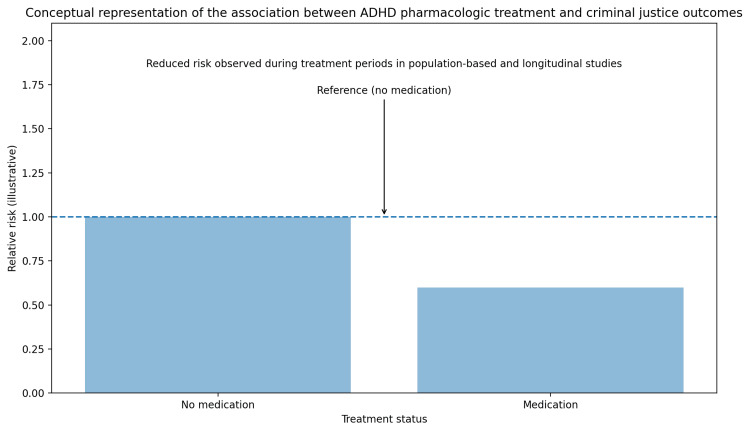
Conceptual representation of the association between ADHD pharmacologic treatment and criminal justice outcomes. This figure provides a conceptual illustration of the reduced risk of behaviors associated with criminal justice involvement during periods of ADHD pharmacologic treatment compared with non-treatment periods. The visual representation is based on patterns consistently reported across population-based and longitudinal studies [12,25-27]. The values depicted are illustrative and do not represent pooled effect sizes, confidence intervals, or estimates from a single dataset. The figure is intended to summarize general trends in the literature rather than provide precise quantitative comparisons. Image credit: Atkins JB. Created using Microsoft Excel and Google Slides. No generative AI tools were used in the creation of this figure. ADHD: Attention-Deficit/Hyperactivity Disorder.

Collectively, these findings suggest that ADHD treatment is associated with reductions in criminal behavior, while emphasizing the need for cautious interpretation. These findings are illustrated in Figure 2.

ADHD in justice-involved populations

ADHD is substantially overrepresented in justice-involved populations. Meta-analytic data indicate that approximately 25-30% of incarcerated individuals meet diagnostic criteria, a prevalence markedly higher than that observed in the general population [14,15].

Beyond prevalence, justice-involved individuals with ADHD often present with significant clinical complexity. Studies of detained youth (N ≈ 1,800) demonstrate high rates of psychiatric comorbidity, including mood disorders, substance use, and other behavioral conditions, frequently in the context of limited prior diagnosis or treatment [17]. Additional analyses (N > 1,000) show elevated rates of suicidal behavior and other adverse mental health outcomes, highlighting the compounded risk profile within this population [18].

These findings suggest that justice-involved populations represent a concentrated group of individuals with ADHD and co-occurring psychiatric conditions, many of whom were not identified or treated before system involvement. This pattern underscores the importance of early identification, integrated mental health care, and intervention before justice system contact.

Racial and ethnic disparities

Disparities in ADHD diagnosis and treatment remain significant. Minority youth, particularly Black and Hispanic children, are less likely to receive an ADHD diagnosis compared with White peers, even when presenting with similar symptom profiles [21]. In large longitudinal analyses of school-based populations (N > 10,000), Black children demonstrated significantly lower odds of receiving an ADHD diagnosis relative to White children, despite comparable behavioral indicators [21].

National data further demonstrate disparities in both diagnosis and treatment. Minority children have lower rates of ADHD diagnosis and reduced access to pharmacologic treatment in large national samples (N > 60,000), contributing to a higher proportion of untreated ADHD in these populations [22,23]. Additional analyses of elementary school populations confirm that sociodemographic factors, including race, ethnicity, and socioeconomic status, influence both identification and treatment patterns [24].

The mechanisms underlying these disparities are multifactorial and operate across clinical, structural, and social domains. Clinician-level factors may include differences in diagnostic thresholds, implicit bias, and variability in the interpretation of behavioral symptoms. Structural factors, such as insurance coverage, access to specialty care, geographic availability of services, and transportation barriers, may limit evaluation and treatment. Social and cultural factors, including language barriers, trust in healthcare systems, prior experiences with discrimination, and differences in knowledge or perceptions of ADHD, may further influence care-seeking behavior and engagement with treatment. School-based referral patterns and disciplinary practices may also contribute to differential identification across populations.

In addition to disparities in access, differences in treatment quality, continuity, and follow-up may further contribute to unequal outcomes. Socioeconomic factors frequently intersect with race and ethnicity, influencing both access to care and the stability of treatment over time.

These disparities are consistent with broader patterns of disproportionate minority contact within the justice system, where minority youth are more likely to experience system involvement at multiple stages [20]. When considered together, these findings suggest that disparities in ADHD diagnosis and treatment may contribute to downstream disparities in justice system involvement, although these relationships are shaped by multiple interacting factors.

Clinical and systems-level considerations

The relationship between ADHD and criminal justice involvement has important implications for clinical practice, particularly in primary care and pediatric settings, where ADHD is often first identified. Early recognition and longitudinal management represent critical opportunities for intervention. Clinical guidelines emphasize comprehensive evaluation, including assessment of comorbid conditions, functional impairment, and environmental context [4]. However, variability in diagnostic practices and treatment continuity persists across healthcare systems, contributing to inconsistent symptom management during key developmental periods.

Effective ADHD management requires sustained clinical engagement. Pharmacologic treatment has been associated with reductions in behaviors linked to criminal justice involvement, including decreased rates of criminal convictions and substance-related problems [12,26,27]. While these findings do not establish causality, they suggest that consistent treatment, medication adherence, and integration of behavioral interventions may influence behavioral trajectories beyond the clinical setting.

The interface between schools and healthcare systems represents a critical point of intervention. Behavioral symptoms are often first observed in educational settings, yet responses may vary from clinical referral to disciplinary action. In under-resourced environments, behavioral concerns may be addressed through exclusionary discipline rather than evaluation, contributing to disengagement and early system contact [19]. Strengthening coordination between healthcare providers, schools, and families may improve early identification and ensure that behavioral symptoms are addressed through appropriate clinical pathways.

Structural and systemic factors further shape outcomes. Disparities in diagnosis and treatment reflect differences in access to care, insurance coverage, provider availability, and potential bias in clinical decision-making [21-24]. These patterns intersect with broader disparities in justice system involvement, where minority youth are more likely to experience system contact at multiple stages [20]. Addressing these inequities requires attention to both clinical practice and structural barriers that influence access to and continuity of care.

Juvenile justice systems themselves represent a potential point of intervention. High rates of psychiatric disorders, including ADHD, have been documented among detained youth, many of whom have not received prior diagnosis or treatment [17,18]. Integrating mental health screening, treatment, and follow-up within justice settings may offer an opportunity to interrupt cycles of recidivism. More broadly, a systems-based approach that emphasizes early identification, coordinated care across sectors, and equitable access to treatment may reduce the downstream risk associated with untreated ADHD.

## Conclusions

This narrative review synthesizes evidence examining the relationships among ADHD, pharmacologic treatment, racial and ethnic disparities, and juvenile justice involvement. Across cohort and longitudinal studies, ADHD is consistently associated with an increased risk of police contact, court involvement, criminal convictions, incarceration, and shorter time to reoffending. ADHD is substantially overrepresented in justice-involved populations, with approximately 25-30% of incarcerated individuals meeting diagnostic criteria, a prevalence several times higher than that observed in the general population. Evidence from population-based registry studies further demonstrates that periods of pharmacologic treatment are associated with lower rates of criminal convictions and substance-related problems. Together, these findings reinforce that ADHD has implications beyond academic or behavioral domains and is associated with broader developmental and population-level outcomes.

These associations occur within a broader structural context. Racial and ethnic disparities in ADHD diagnosis and treatment persist across national samples, with Black and Hispanic youth less likely than White peers to receive evaluation and pharmacologic care despite comparable symptom burden, likely reflecting differences in access, referral patterns, and engagement with healthcare systems. These inequities intersect with disproportionate minority contact in the justice system, suggesting that untreated ADHD may represent one modifiable factor among several influencing downstream outcomes. Addressing this pathway requires expanding equitable screening in primary care and school settings, improving access to longitudinal pharmacologic and behavioral care, strengthening coordination across healthcare, educational, and juvenile justice systems, and implementing routine mental health screening in detention settings with clear pathways for treatment and continuity of care. However, the evidence base remains limited by observational study designs, heterogeneity across studies, and limited juvenile-specific data. Future research is needed to better define causal pathways and evaluate integrated interventions across systems.
